# Platelet Aggregation Before Aspirin Initiation in Pediatric Patients With Congenital Heart Disease at High Risk of Thrombosis

**DOI:** 10.3389/fcvm.2022.813190

**Published:** 2022-07-13

**Authors:** Zhong-Yuan Lu, Zhi-Yuan Zhu, Ju-Xian Yang, Yu-Zi Zhou, Ya-Zhou Jiang, Wei Wei, Xu Wang, Shou-Jun Li

**Affiliations:** ^1^Pediatric Intensive Care Unit, Pediatric Cardiac Center, Fuwai Hospital, National Center for Cardiovascular Diseases, Chinese Academy of Medical Science and Peking Union Medical College, Beijing, China; ^2^Department of Cardiac Surgery, Pediatric Cardiac Center, Fuwai Hospital, National Center for Cardiovascular Diseases, Chinese Academy of Medical Science and Peking Union Medical College, Beijing, China

**Keywords:** platelet aggregation, congenital heart disease, children, basal status, influencing factors

## Abstract

**Background:**

Aspirin following unfractionated heparin is the most common anticoagulation strategy for pediatric patients who experienced cardiac surgery at high risk of thrombosis. The platelet aggregation test is the golden method to evaluate the aspirin effect on platelet function. However, the platelet aggregation basal status before postoperative aspirin initiation and the related clinical influencing factors hasn't been investigated systemically in this population.

**Methods:**

In a prospective cohort of 247 children, arachidonic acid-induced platelet aggregation (PAG-AA) was measured by means of light transmission aggregometry (LTA) before the first dose of aspirin after cardiac surgical procedure and the perioperative variables were also collected. Distribution of this population's PAG-AA basal status was described. Univariate and multivariate logistic regression analysis were performed to identify the main influencing factors of PAG-AA.

**Results:**

The median time of aspirin administration was 2 (1–27) days after surgery and the corresponding median value of basal PAG-AA was 20.70% (1.28–86.49%), with 67.6% population under 55% and 47.8% population under 20%. Patients undergoing cardiopulmonary bypass (CPB) had a significantly lower basal PAG-AA than those without (30.63 ± 27.35 vs. 57.91 ± 27.58, *p* = 0.013). While patients whose test done within 3 days after CPB had a significantly lower PAG-AA than those out of 3 days (25.61 ± 25.59 vs. 48.59 ± 26.45, *p* = 0.001). Univariate analysis implied that the influencing factors of the basal PAG-AA including CPB use, test time point, cyanosis, and platelet count. Multivariate regression analysis indicated that only CPB use, test time point, and platelet count were the main independent influencing factors for the basal PAG-AA.

**Conclusion:**

The majority of children have impaired basal platelet aggregometry responses before postoperative aspirin initiation. The main influencing factors are CPB use, test time point, and platelet count. To establish the platelet aggregometry baseline prior to commencement of aspirin therapy, testing should be performed 3 days later following the procedure when effect of CPB is basically over.

## Introduction

Children at high risk of thrombosis with congenital heart disease are a large and important population in need of antithrombotic therapy (1, 2). Aspirin is the most common and economical antithrombotic drug for them with the mechanism of platelet aggregation inhibited by inactivating the cyclooxygenase (3–6). However, the clinical results are still not satisfactory with the current treatment strategy, as the incidence of thrombosis and bleeding can up to 10–30% (7, 8), respectively. Based on the platelet function assessment, aspirin dosage adjustment may be a better method to improve the clinical outcomes. While, light transmission aggregometry (LTA) is considered to be the gold standard test for platelet function monitoring (9, 10). Nevertheless, current researches on platelet aggregation are mainly focused on adults while few studies are applied in children. Studies on the basal state of platelet aggregation in children with congenital heart conditions at high risk of thrombosis before the initiation of aspirin have not been systemically reported. Thus, our study is to explore the basal state of platelet aggregation and the associated main influencing clinical factors in this population. This baseline establishment may not only supply evidences to an individualized aspirin dosage prescription, but also can give a reference to accurately evaluate the following aspirin effect.

## Methods

### Patients and Study Design

A prospective cohort observational study was carried out in Fuwai Hospital from January 1, 2020 to December 31, 2020. All children at high risk of thrombosis with congenital heart disease in our Pediatric Cardiac Surgery Center who was to receive primary antithrombotic therapy with aspirin after cardiac surgery were enrolled in this study. Children at high risk of postoperative thrombosis were identified as the following situations: (1) implantation of prosthetic material into the circulation (aortopulmonary shunt, Glenn procedure, and intracardiac baffles); (2) coronary artery reconstruction (arterial switch operation, coronary artery unroofing or re-implantation procedures); (3) pulmonary vein reconstruction (correction of total anomalous of pulmonary vein connection, and correction of pulmonary vein stenosis); (4) complex valve repair/replacement. Exclusion criteria included: (1) documented thrombosis before initiation of aspirin therapy; (2) had medication interfering with coagulation or platelet function prior to surgery; (3) unable to collect blood sample. This study was approved by the Ethics Committee of Fuwai Hospital (2020-1311). A written informed consent was obtained from both parents.

### Anesthesia, Bypass, and Surgical Technique

The management of anesthesia, bypass, and surgery was according to the standard protocol carried in our unit. Midazolam, cisatracurium besilate, and ketamine were used for induction of anesthesia. Maintenance of anesthesia included isoflurane and fentanyl. A total of 400 units kg^−1^ unfractionated heparin was given before the cardiopulmonary bypass (CPB) circuit inserted. During CPB, activated clotting time above 480 s was maintained through additional heparin doses. Heparin was neutralized with protamine sulfate after CPB. The CPB circuit was primed with crystalloids, human albumin 20%, and packed red blood cells aiming at a target hematocrit of 27–30% during CPB. Rectal temperature (28–35°C) was decided by the surgeon depending on the type of surgery. Weaning off CPB was performed after rewarming to 36°C using dopamine and milrinone. During the procedures, autologous blood transfusion strategy was applied. Additional red blood cell (RBC) transfusions were given when the hematocrit was <25% while on CPB and <30% after CPB. For neonates, platelet was firstly transfused if there was a diffuse bleeding when heparin was neutralized and surgical hemostasis was completed after CPB. For other patients, platelet was given in case of persisting bleeding with platelet count < 100 × 10^9^/L. Fresh-frozen plasma (FFP) was transfused in case of clinical bleeding and prothrombin time > 40% above control.

### Anticoagulation Strategy and the Basal Platelet Aggregation Test

Unfractionated heparin (UFH) was initiated when there was no bleeding tendency after cardiac surgical procedure, usually on the late time of the day that patients returned to the ICU with a dose of 10–20 U/kg/h. The target value of the activated partial thromboplastin time (APTT) was maintained at 55–70 s. After ventilator was weaned, UFH was switched to aspirin through oral or nasogastric tube. Blood sample was obtained just before the first administration of aspirin at 6 am with a standard procedure (from a central venous catheter or an arterial cannula discarding the first 6 ml at each blood sampling and drawn into 3.2% sodium citrate tubes). Subsequently, the sample was taken to our central laboratory within 20 min and centrifuged at 200 *g* for 10 min at room temperature to prepare platelet-rich plasma (PRP). Then the PRP was moved into a special plastic tube for storage and for the following test of platelet aggregation (PAG) by the automatic analyzer LBY-NJ4A (PRECIL, Beijing, China) with light transmission aggregometry. The 0.5 mmol/L arachidonic acid (AA) was used as the inducer (11–13). The platelet count in PRP was not standardized prior to the aggregometry according to our laboratory guiding in this test and previous studies (14, 15). Platelet count above 100 × 10^9^ per liter would yield valid results. All the processes were finally finished within 2 h since blood was collected. Results of the sample at this point were taken as the basal PAG-AA. Because no result cutoffs for pediatrics with this method have been published for reference, we adopted the standard from our hospital's central laboratory, which referred to the manufacturer's values for healthy adults. The specific information was as follows: PAG-AA ≥ 55% was considered to be normal, <55% was reduced, and ≤20% was considered to be severely reduced.

### Clinical Data Collection and Thrombosis Determination

The pre, intra, and postoperative clinical data of all the patients were collected. Preoperative data included gender, age, weight, specific diagnosis of congenital heart disease, and cyanosis or not. Intraoperative data included whether CPB was performed, CPB time, aortic cross-clamp time, and surgical procedure. Early postoperative data included PAG-AA, the time point of blood sampling, platelet count (blood sample obtained at the same time of PAG-AA), hepatic, and renal function in the same period (blood sample obtained within 1 day of PAG-AA test). The criteria of thrombosis were defined as: (1) Clinical thrombotic events, such as stroke, limb ischemia, and shunt thrombosis; (2) Imaging evidence of thrombus, peripheral, and cardiac evidence were both included, no matter from ultrasound or cardiac catheterization. All patients were evaluated for thrombosis on the day before aspirin initiation.

### Statistical Analysis

The Software SPSS 23.0 for Windows was used for statistical analysis. Firstly, normality test was performed for all the continuous variables. Data with normal distribution were expressed as mean ± standard deviation (X ± s) and *t*-test was performed when univariate analysis. Data with non-normal distribution were summarized by median (minimum value, maximum value) and rank sum test was performed when univariate analysis. All the categorical data were tested by chi-square test. The correlation was assessed with the Pearson's test. Finally, on the basis of univariate analysis, multivariate logistic regression analysis was conducted on the possible influencing factors for the reduction of PAG-AA. The *p* < 0.05 was considered statistically significant. Because of the exploratory nature of the study, no power calculation was performed.

## Results

### Patients' Characteristics and Basal PAG-AA Status

Within the study period, a total of 258 children at high risk of thrombosis underwent cardiac surgery in our center and was deemed to receive the primary antithrombotic therapy with aspirin, while the informed consent was obtained from only 252 patients' parents. Then 2 patients were found to develop the inferior vena cava thrombus from echocardiogram imaging, 1 with shunt conduit thrombosis and 1 with limb ischemia before aspirin administration. Additionally, another one patient failed to get blood before aspirin administration. In the end, 247 patients' characteristics were used for the subsequent analysis. All the demographic and surgical data were shown in Table 1. Totally, 106 patients with cyanotic congenital heart disease 141 with acyanotic, 226 patients underwent surgeries with CPB, the other 21 patients without (17 aortopulmonary shunt procedure and 4 bidirectional Glenn procedure). Approximately 18% of patients received platelet transfusion during surgery because of the diffuse bleeding when heparin was neutralized and surgical hemostasis was completed after CPB. Just 2 patients received additional platelet in the early ICU days prior to aspirin initiation. All the patients had a platelet count > 100 × 10^9^/L when aspirin initiation.

**Table 1:** Demographics and surgical characteristics.

**Characteristic**	**Value**
Total number (*n*)	247
Male/female (*n*)	147/100
Median age at surgery (m)	13 (0.4–72)
Mean weight (kg)	10.2 ± 4.9
Surgical procedure (*n*)	
mitral valvuloplasty (Annuloplasty ring)	68
Correction of PAPVC	21
Correction of TAPVC	16
PAVSD repair	16
CAVSD repair	7
Pulmonary artery reconstruction	31
Bidirectional Glenn	13
Aortopulmonary shunt	21
Arterial switch operation	10
ALCAPA coronary artery reimplantation	7
Coronary artery unroofing	4
Correction of pulmonary vein stenosis	4
Vena cava reconstruction	5
Senning procedure	7
Rastelli procedure	7
Other complex valve repair	10

*PAPVC, partial anomlous of pulmonary venous connection; TAPVC, total anomlous of pulmonary venous connection; PAVSD, partial atrioventricnlar septal defect; CAVSD, complete atrioventricnlar septal defect; ALCAPA, anomalous origin of the left coronary artery from the pulmonary artery*.

**Figure 1:**
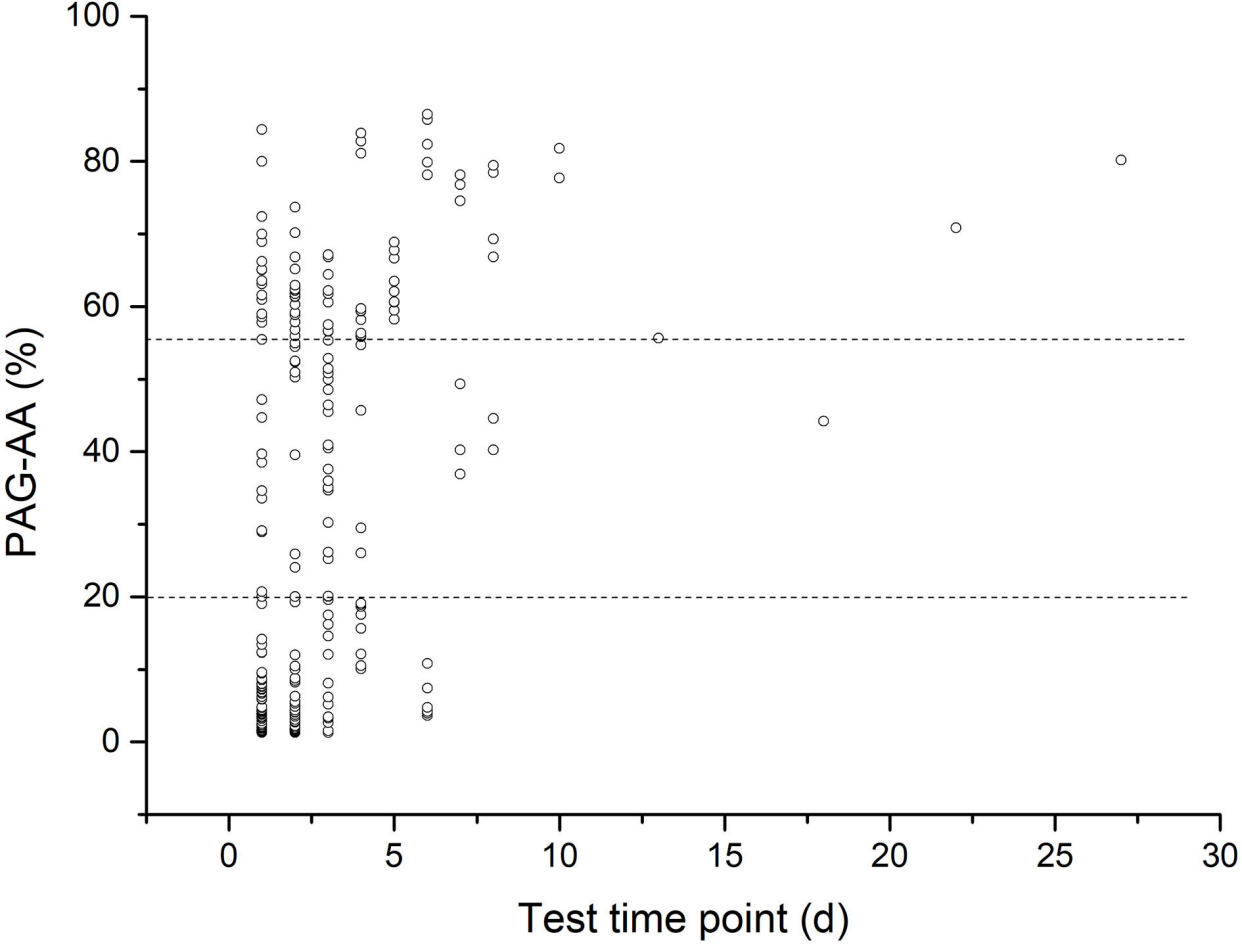
The scatterplot of basal arachidonic acid- induced platelet aggregation (PAG-AA) distribution.

The median time point of aspirin initiation was 2 (1–27) days after surgery. Because the basal PAG-AA test was done at the same day of the first aspirin dose administration and just before it, so this was also the median time point of basal PAG-AA test. As for the results, median value of PAG-AA was 20.70% (1.28–86.49%), with 67.6% population below normal, 47.8% population severely reduced (below 20%). The overall distribution is shown in Figure 1. All patients were in stable condition with no bleeding tendency on the day of testing, and then aspirin was administered either orally or *via* nasogastric tube.

In terms of different population groups, patients under CPB had a significantly lower basal PAG-AA than the patients without (30.63 ± 27.35 vs. 57.91 ± 27.58, *p* = 0.013). CPB group with the mean value below normal and the off-CPB group had a mean value within normal range, respectively. Tests also showed that there was a profound difference of basal PAG-AA between the cyanotic patients and the acyanotics. It was remarkable higher in the cyanotic group (42.67 ± 28.00 vs. 25.87 ± 26.40, *p* = 0.004). Furthermore, patients who had received platelet transfusion during the procedure had significant higher basal PAG-AA than those without (43.98 ± 31.07 vs. 24.90 ± 25.14, *p* = 0.048).

### The Impact of Test Time Point on the Basal PAG-AA

A Pearson correlation analysis was carried out between these two continuous variables. It showed that the basal PAG-AA had a significant positive correlation with the time point of testing (*r* = 0.376, *p* < 0.001).

**Figure 2:**
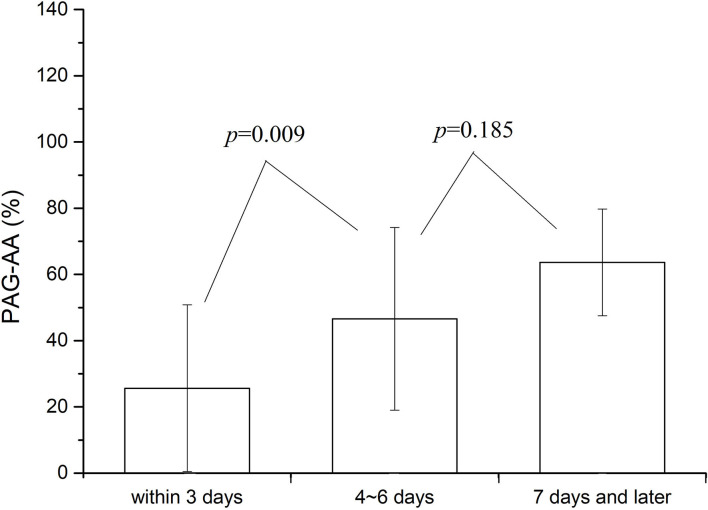
Comparison of basal PAG-AA at different test time point.

A more detailed statistical analysis was also made. Summaries showed that among all the patients, 186 cases were tested within 3 days after surgery (the time point of blood collection was within 72 h of operation), 43 cases at 4–6 days after surgery, and the rest 18 cases at 7 days or later. The value of PAG-AA was compared among the patients at the three different test stages. Results were shown in Figure 2. No significant difference was found in PAG-AA between patients whose test were done at 4–6 and 7 days or more after surgery (46.56 ± 28.03 vs. 63.62 ± 18.38, *p* = 0.185). But there was a significant difference on the basal PAG-AA when comparison between the patients whose test done within 3 days and beyond 3 days after surgery. Test within 3 days was much lower (25.61 ± 25.59 vs. 48.59 ± 26.45, *p* = 0.001). This difference was even more pronounced when CPB patients were analyzed separately. However, no difference was there for the patients without CPB. Consequently, we may make a conclusion that the test time point play an impact mainly on the patients under CPB and this impact was most obvious within 3 days after the operation.

### Univariate Analysis and Multivariate Logistic Regression Analysis for the Basal PAG-AA

Patients were divided into the decreased and normal basal PAG-AA group according to the test. The Univariate analysis was performed to identify the potential clinical influential factors for PAG-AA (Table 2). The involving factors included the previously discussed factors above and some other possible factors such as small age (≤1 year), low weight (≤5 kg), hepatic and renal function, contemporaneous platelet count, and the pumping dose of heparin just before aspirin initiation (U/kg/h). Results showed that besides CPB use, cyanosis and test time point, platelet count in the same stage is another influencing factor for basal PAG-AA. It was significantly higher in the normal PAG-AA group (234.4 ± 51.9 vs. 164.9 ± 49.2, *p* = 0.010). Other factors were found having no prominent impact on the basal PAG-AA (all *p* > 0.05).

**Table 2:** Univariate analysis for decreased basal arachidonic acid-induced platelet aggregation (PAG-AA).

	**Reduced PAG-AA**	**Normal PAG-AA**	**t/χ^2^**	***P***
CPB (*n*)				
Yes	162	64		
No	5	16	20.108	<0.001
Cyanosis (*n*)				
Yes	52	54		
No	115	26	29.193	<0.001
Test within 3 days (*n*)				
Yes	144	42		
No	23	38	33.085	<0.001
Weight ≤ 5 kg (*n*)				
Yes	15	11		
No	152	69	1.306	0.253
Age ≤ 12 m (*n*)				
Yes	76	46		
No	91	34	3.111	0.078
Pumping heparin dose	8.4 ± 3.2	10.6 ± 5.4	−0.357	0.684
(U/kg/h)				
PLT (× 10^9^/L)	164.9 ± 49.2	234.4 ± 51.9	−2.796	0.010
Duration of CPB (min)	119.9 ± 31.0	153.5 ± 57.1	−1.719	0.100
Time of ACC (min)	78.7 ± 26.5	85.0 ± 47.7	−0.256	0.813
ALT (U/L)	19.9 ± 8.6	24.5 ± 16.3	−0.667	0.514
AST (U/L)	124.2 ± 65.6	138.5 ± 17.7	−0.300	0.768
Cr (umol/L)	42.4 ± 10.3	39.5 ± 2.1	0.395	0.697

*CPB, cardiopulmonary bypass; ACC, aortic cross clamping; PLT, platelet; ALT, alanine aminotransferase; AST Aspartate Transaminase; Cr, creatinine*.

To determine the independent influencing factors for basal PAG-AA, a further analysis of multivariate logistic regression was done. All the potential factors (all the *p*-value ≤ 0.1) found in the univariate analysis were taken into the regression equation. Thus, duration of CPB, small age (≤1 year) was also included. Results were shown in Table 3. CPB use, the time point of testing and platelet count were the independent clinical influencing factors for reduced basal PAG-AA eventually. Age ≤1 year, cyanosis, and duration of CPB had no significant impact on the basal PAG-AA value.

**Table 3:** Logistic multivariate regressive analysis for decreased basal PAG-AA.

**Influential factors**	**B**	**S.E**.	**Wal**	***P***	**Exp (β)**
CPB use	1.343	0.952	3.087	0.032	3.714
Cyanosis	−0.702	0.527	1.467	0.244	0.512
Test time point	1.413	0.516	5.783	0.016	4.179
Age ≤ 12 m	−0.347	0.559	0.376	0.477	0.635
PLT	0.162	0.102	5.265	0.022	0.986
Duration of CPB	0.233	0.113	1.572	0.154	0.164

*CPB, cardiopulmonary bypass; PLT, platelet*.

## Discussion

A sequential anticoagulation treatment that aspirin following unfractionated heparin is a common and conventional strategy for the pediatric postoperative cardiac patients at high risk of thrombosis (1, 7, 16, 17). Platelet function test by platelet aggregation is the gold standard method to evaluate the aspirin effect (8, 18, 19). Data about the platelet function at preoperation in these patients have been investigated in many centers (11, 20, 21). However, limited data (8, 18, 19, 22) can be found focusing on the basal state of platelet function just before the postoperative aspirin initiation. Thus, we carried out this study which included almost all types of CHD procedures requiring aspirin anticoagulation to systemically explore the basal status of PAG-AA.

Our study found that majority of the patients had a decreased PAG-AA just before aspirin initiation. This tendency was same as Truong and his colleagues found, although their sample was small (8). Simultaneously, it also demonstrated that the platelet function impairment is common in patients after cardiac surgery, consistent with the previous results obtained by other testing method (23, 24). On the other hand, it also indicates that the timing of aspirin start is appropriate while platelet function is still inhibited but with no bleeding tendency. In addition, it is worth mentioning that although about half of the basal PAG-AA was in very low level (≤20%), no obvious bleeding was observed and the aspirin therapy was followed. No additional intervention such as platelet supplement should be done just according to this test.

Cardiopulmonary bypass is an important influencing factor for basal PAG-AA. The mean value of basal PAG-AA is much lower in CPB group. In our study, more than 70% of the basal PAG-AA was normal when without CPB. Conversely, more than 70% was below normal when CPB was used. Basal PAG-AA had a significant negative correlation with the CPB (*r* = −0.455, *p* < 0.001). This is basically consistent with the results of studies in adults (23), although a different test method was applied. However, the duration of CPB didn't play a significant impact on the value of basal PAG-AA. No close correlation was found between the two variates in our study (*r* = 0.216, *p* = 0.175), which means that even a short time of CPB can result in a severely decreased PAG-AA. This was different from the study by Bønding Andreasen et al. (20). Blood exposure to the non-endothelialized lining of the cardiopulmonary bypass circuit, flow shear stress, hemodilution, hypothermia, and heparin administration, all contributed to the consumption and destruction of the platelet, inducing platelet dysfunction (15).

In our study, test time point of basal PAG-AA is another independent influencing factor for basal PAG-AA. But why test time point can lead an impact on the basal PAG-AA in this population? In our detailed analysis, we found that the time effect only occurred in patients under CPB and no PAG-AA difference was observed in patients without CPB as time changed. Therefore, we may make a conclusion that the time effect actually reflect the length of CPB after effect. The platelet function impairment caused by CPB will last at least for 3 days for most patients under CPB. Hofer et al. also found the impact of CPB on platelet function as time passed (21) and they noticed this effect would last for 5 days before the platelet function recovered to the preoperative levels. Comparing with them, our study showed a shorter effect of CPB on platelet function with just 3 days. This difference may be interpreted by the different technique of CPB in different centers and also by the analysis deviation from longitudinal to cross-sectional study. Whatever, considering the CPB after effect, we suggest a PAG-AA test beyond the early 3 postoperative days as the reference benchmark.

The platelet count is also an independent clinical influencing factor. Although all the platelet count was above 100 × 10^9^/L in a normal state, which seems to be in the same level in our study, it actually brought different levels of PAG-AA just due to the different numbers. Some other studies also found this impact in adult patients (25, 26). More platelet count was always associated with increased PAG-AA. But the correlation intensity was not so strong when Pearson analysis was done between these two continuous variables (*r* = 0.361, *p* < 0.001). This supports the idea that measurements of platelet count alone are insufficient for the estimation of platelet function after pediatric cardiac surgery.

Our study showed that cyanosis was not an independent influencing factor for basal PAG-AA. Although in univariate analysis, basal PAG-AA was significantly higher in cyanotic patients than that of the acyanotics; eventually it was found that cyanosis had no significant effect on basal PAG-AA in multivariate analysis. This was consistent with most of the findings in associated investigations, although different methods applied (20, 27–29). However, Hofer et al. noticed both a higher pre- and postoperative PAG-AA in cyanotic patients which represented better platelet function theoretically. Actually, more bleeding was noticed in cyanotic patients (21). We speculated that this paradoxical phenomenon may be due to the deviation from their small sample size and lack of multivariate analysis.

## Limitations

There are still some shortcomings in this study. First of all, since this study is a prospective cohort study, the results may naturally be affected by differences in selected samples. In this study, on-CPB and off-CPB patients' sample size did not match. The sample size of off-pump patients is relatively small, and there may be bias on the analysis of the impact of basal PAG-AA. Secondly, we also recorded platelet count, liver and kidney function and other data during the same period, but since almost all of them were normal, we didn't find a positive or negative impact of these factors, which may need further discussion. Finally, we only observed and evaluated the basal PAG-AA just before the initiation of aspirin; the changes after the use of aspirin are still to be discussed in the future.

## Conclusion

For pediatric patients at high risk of thrombosis following surgical procedures with congenital heart disease, the majority has impaired light transmittance platelet aggregometry responses induced by arachidonic acid prior to commencement of aspirin therapy. The main independent clinical influencing factors of this basal PAG-AA are CPB use, test time point and platelet count. CPB involvement, test time point within 3 days of the procedure and lower platelet count are associated with reduced PAG-AA. Cyanosis and small age have no significant impact on that. To establish the platelet aggregometry baseline prior to commencement of aspirin therapy, testing should be performed 3 days later following the procedure when effect of CPB is basically over.

## Data Availability Statement

The original contributions presented in the study are included in the article/supplementary material, further inquiries can be directed to the corresponding author.

## Ethics Statement

This study was approved by the Ethics Committee of Fuwai Hospital (2020-1311). Written informed consent to participate in this study was provided by the participants' legal guardian/next of kin.

## Author Contributions

Z-YL and Z-YZ designed the study, collected and analyzed the data, and they were major contributors in writing the manuscript. Y-ZZ, Y-ZJ, and WW recruited patients, analyzed, and interpreted the clinical data. XW designed the study, recruited patients, analyzed the data, and revised of the manuscript. J-XY and S-JL helped to perform the study and gave important constructive suggestions. All authors read and approved the final manuscript.

## Funding

This work was supported by the Clinical Translational Medicine Research Foundation from Chinese Academy of Medical Sciences (Grant Nos: 2019XK320053 and 2020-I2M-C&T-B-012).

## Conflict of Interest

The authors declare that the research was conducted in the absence of any commercial or financial relationships that could be construed as a potential conflict of interest.

## Publisher's Note

All claims expressed in this article are solely those of the authors and do not necessarily represent those of their affiliated organizations, or those of the publisher, the editors and the reviewers. Any product that may be evaluated in this article, or claim that may be made by its manufacturer, is not guaranteed or endorsed by the publisher.
